# Increased bystander intervention when volunteer responders attend out-of-hospital cardiac arrest

**DOI:** 10.3389/fcvm.2022.1030843

**Published:** 2022-11-04

**Authors:** Christian Gantzel Nielsen, Fredrik Folke, Linn Andelius, Carolina Malta Hansen, Ulla Væggemose, Erika Frischknecht Christensen, Christian Torp-Pedersen, Annette Kjær Ersbøll, Mads Christian Tofte Gregers

**Affiliations:** ^1^Copenhagen Emergency Medical Services, Copenhagen University Hospital, Copenhagen, Denmark; ^2^Department of Clinical Medicine, University of Copenhagen, Copenhagen, Denmark; ^3^Department of Cardiology, Copenhagen University Hospital – Herlev and Gentofte, Copenhagen, Denmark; ^4^Department of Anaesthesiology, Copenhagen University Hospital – Herlev and Gentofte, Copenhagen, Denmark; ^5^Department of Cardiology, Rigshospitalet, University of Copenhagen, Copenhagen, Denmark; ^6^Department of Research and Development, Prehospital Emergency Medical Services, Central Denmark Region, Aarhus, Denmark; ^7^Department of Clinical Medicine, Aarhus University, Aarhus, Denmark; ^8^Centre for Prehospital and Emergency Research, Department of Emergency Medicine and Trauma Center, Aalborg University Hospital, Aalborg, Denmark; ^9^Department of Clinical Medicine, Aalborg University Hospital, Aalborg, Denmark; ^10^Prehospital Emergency Services, North Denmark Region, Aalborg, Denmark; ^11^Department of Cardiology, Copenhagen University Hospital – North Zealand, Copenhagen, Denmark; ^12^Department of Public Health, University of Copenhagen, Copenhagen, Denmark; ^13^National Institute of Public Health, University of Southern Denmark, Copenhagen, Denmark

**Keywords:** out-of-hospital cardiac arrest, volunteer responders, bystander interventions, cardiopulmonary resuscitation, defibrillation

## Abstract

**Aim:**

The primary aim was to investigate the association between alarm acceptance compared to no-acceptance by volunteer responders, bystander intervention, and survival in out-of-hospital cardiac arrest.

**Materials and methods:**

This retrospective observational study included all suspected out-of-hospital cardiac arrests (OHCAs) with activation of volunteer responders in the Capital Region of Denmark (1 November 2018 to 14 May 2019), the Central Denmark Region (1 November 2018 to 31 December 2020), and the Northern Denmark Region (14 February 2020 to 31 December 2020). All OHCAs unwitnessed by Emergency Medical Services (EMS) were analyzed on the basis on alarm acceptance and arrival before EMS. The primary outcomes were bystander cardio-pulmonary resuscitation (CPR), bystander defibrillation and secondary outcome was 30-day survival. A questionnaire sent to all volunteer responders was used with respect to their arrival status.

**Results:**

We identified 1,877 OHCAs with volunteer responder activation eligible for inclusion and 1,725 (91.9%) of these had at least one volunteer responder accepting the alarm (accepted). Of these, 1,355 (79%) reported arrival status whereof 883 (65%) arrived before EMS. When volunteer responders accepted the alarm and arrived before EMS, we found increased proportions and adjusted odds ratio for bystander CPR {94 vs. 83%, 4.31 [95% CI (2.43–7.67)] and bystander defibrillation [13 vs. 9%, 3.16 (1.60–6.25)]} compared to cases where no volunteer responders accepted the alarm.

**Conclusion:**

We observed a fourfold increased odds ratio for bystander CPR and a threefold increased odds ratio for bystander defibrillation when volunteer responders accepted the alarm and arrived before EMS.

## Introduction

During the last decade, a strategy of activating volunteer responders to increase bystander cardiopulmonary resuscitation (CPR) and early defibrillation with automated external defibrillators (AEDs) has been implemented world-wide with positive results and increasing interest from both the general public and professionals (1–11). Globally, survival following out-of-hospital cardiac arrest (OHCA) ranges from 2 to 11% with great variations (12, 13). Experiences from US casinos and Copenhagen Airport have reported survival rates between 74 and 100% within the subgroup of OHCAs with initial shockable heart rhythm (14, 15). These findings imply a large potential to increase survival if defibrillation can be achieved within minutes from OHCA and support the continued development and implementation of bystander engaging initiatives such as volunteer responder programs (16), as recommended by both the American Heart Association and the European Resuscitation Council (17, 18). However, there are currently great variations in design and reporting within volunteer responder systems worldwide and knowledge regarding factors that influence whether volunteer responders accept the alarm are scarce. Likewise, little is currently known about the relation between alarm acceptance and the volunteer responder arriving and assisting at the site of OHCA. Information in these areas is important to further understand and improve volunteer responder systems. In this study, we aimed to investigate bystander interventions and 30-day survival when volunteer responders accepted the alarm compared to OHCAs where no volunteer responders accepted the alarm. Our primary analysis was in the group where volunteer responders reported arriving before Emergency Medical Services (EMS). Secondary analysis was done where volunteer responders reported arriving at the scene of OHCA and finally we compared OHCAs where at least one volunteer responder accepted the alarm compared with OHCAs where no volunteer responders accepted the alarm (irrespective of when or if they arrived at the scene and before EMS). These secondary analyses were included to compare our volunteer responder programs to other international programs with less complete data and/or missing data about volunteers’ time of arrival. We hypothesized that when a volunteer responder accepted the alarm and arrived before EMS compared to not it was associated with increased bystander interventions (CPR and defibrillation) and 30-day survival. Furthermore, we hypothesized that a larger proportion of alarms not accepted occurred during nighttime and in rural areas.

## Materials and methods

### Study setting

This observational study with prospective data collection included OHCAs with activation of volunteer responders in the Capital Region of Denmark (1 November 2018 to 14 May 2019), the Central Denmark Region (1 November 2018 to 31 December 2020), and the Northern Denmark Region (14 February 2020 to 31 December 2020). Due to an ongoing randomized controlled trial, data collection for the Capital Region does not include patients after 14 May 2019. All three included regions consist of both urban, suburban, and rural areas and covers approximately 23,554 km^2^ (≈55% of Denmark) and inhabits 3.75 million people (≈64% of the total population).

#### Emergency Medical Services

All included regions are served by a two-tiered EMS system consisting of an ambulance and a physician-staffed unit which are dispatched in case of suspected OHCA. The three regions have separate and independent dispatch centers but follow the same standardized protocol in the event of suspected OHCA. In addition, emergency dispatchers perform telephone-guided CPR and assistance with information on accessing the nearest available AED. In Denmark, a national AED registry was established in 2007 and now contains >21,000 publicly available registered AEDs. The AED registry is linked to all dispatch centers with information on opening hours, accessibility, and global position system location. All EMS dispatchers in Denmark can activate volunteer responders in case of suspected OHCA to assist with CPR and acquisition of a nearby AED. Volunteer responders can be activated simultaneously with the ambulance but usually the activation occurs 30−60 s later as the dispatcher needs to make sure that the surroundings are safe for volunteer responders to attend (6). In this study, all interventions before the arrival of EMS are referred to as bystander interventions. It is not possible to differentiate between interventions performed by random bystanders and volunteer responders.

#### The Danish volunteer responder program

The program was first implemented in the Capital Region of Denmark in September 2017 with other regions gradually following resulting in full national coverage by May 2020. The program is based on volunteers willing to assist in case of OHCA. The purpose of the program is to improve bystander intervention prior to the arrival of EMS to ultimately increase the chances of survival (19). In case of a nearby OHCA, volunteers are activated *via* a smartphone application, and when activated, the volunteer responder can either accept or reject the alarm. When the alarm has been accepted, the volunteer responder is guided either directly to the site of OHCA or *via* an AED registered in the national AED registry. The nearest 20 volunteer responders within 1,800 m of the potential OHCA are activated. We have previously demonstrated that approximately 50% of the volunteer responders react to the alarm whereof roughly 50% accept the alarm (overall acceptance rate 25−30%) (6, 20). All volunteer responders must be ≥18 years of age to register with the program. Previous experience or certified CPR training are not required to register but are highly recommended. After 90 min from dispatch, volunteer responders are asked to complete a questionnaire about their participation and experiences related to their mission. Emergency dispatchers do not activate the volunteer responder system in OHCAs involving suicide, trauma, children <8 years or if OHCA surroundings are deemed unsafe. Previous publications describe the program in more detail (6, 20).

### Study population, groups, and design

All presumed OHCAs, assessed by emergency dispatchers, with volunteer responder activation from the Central, the Capital and North regions of Denmark were identified. Confirmed OHCA was defined as OHCA registered in the Danish Cardiac Arrest Registry, thus we excluded cases (non-OHCAs) not found in the Danish Cardiac Arrest Registry. Further, we excluded OHCAs witnessed by EMS and OHCAs with volunteer responder activation but with no one within range (<1,800 m) of the OHCA. The study population was divided into two groups: one group where at least one volunteer responder accepted the alarm (referred to as “accepted”) and one group where no volunteer responder accepted the alarm (referred to as “not-accepted”). The “not-accepted” group thus includes both rejected and unseen/unanswered alarms. Further, the primary analysis was in the group where volunteer responders reported arriving before EMS in the subsequent questionnaire sent to them. Secondary analysis included OHCAs where at least one volunteer responder accepted the alarm and arrived at the site of OHCA (irrespective of EMS arrival) and OHCAs where at least one accepted the alarm (irrespective of their reported arrival status) both compared to OHCA where no volunteer responders accepted the alarm. This was done in order to compare our data with different volunteer responder programs where arrival status of the volunteer responders is unavailable for scientific reporting. Finally, as Supplementary data we provided a comparison of patients according to initial shockable rhythm.

### Study parameters and data sources

Variables related to the OHCA originate from the Danish Cardiac Arrest Registry which includes time and date of OHCA, latitude and longitude of OHCA, home or public location, age, sex, witnessed status, initial shockable heart rhythm [ventricular fibrillation (VF) or pulseless ventricular tachycardia (pVT)], bystander CPR, bystander defibrillation, EMS response time, EMS defibrillation, ROSC, and 30-day survival. Information regarding volunteer responders originated from the Volunteer Responder Application Server (local register) and includes geographical locations, app-interactions when accepting or declining alarms, sex, and age. Population density estimates at the OHCA site were based on the municipal population density and were stratified according to the EUROSTAT degree of urbanization system (DEGURBA) producing a three-layered population density stratification (low, intermediate, and high) (21).

### Study outcomes

The primary outcomes for this study were bystander CPR, bystander defibrillation, and secondary outcome was 30-day survival.

### Statistical analysis

Categorical variables were presented as proportions and percentages and continuous variables were presented as medians with interquartile range (IQR). A logistic regression analysis was performed to investigate association between exposure (volunteer responders accepting the alarm and their arrival status) and primary outcome variables (bystander CPR, defibrillation, and 30-day survival). Furthermore, we performed a multivariable logistic regression analysis to adjust for identified confounders. We used Direct Acyclic Graphs to determine potential confounders affecting both the exposure and outcomes, Supplementary Figures 1–3. Only time of day (of the OHCA) and the degree of urbanization were deemed confounders which we adjusted for in the logistic regression analysis. Statistical analysis was carried out in SAS Enterprise Guide version 7.1 (SAS Institute Inc., Cary, NC, United States) and RStudio version 1.2.1335 (22).

### Ethical and legal approval

Data were obtained and stored according to the Danish Data Protection Agency (P-2021-670 and P-2021-82). According to Danish Law register studies do not require ethical approval. The study was approved by the Danish Safety Authority (3-3013-2721/1). At registration, volunteer responders give permission to be contacted by the research team if necessary. Volunteer responders consent not to disclosure any private information in relation to OHCA alarms and resuscitation attempts. Volunteer responders can withdraw from the program at any time and simultaneously withdraw their consent.

## Results

**FIGURE 1:**
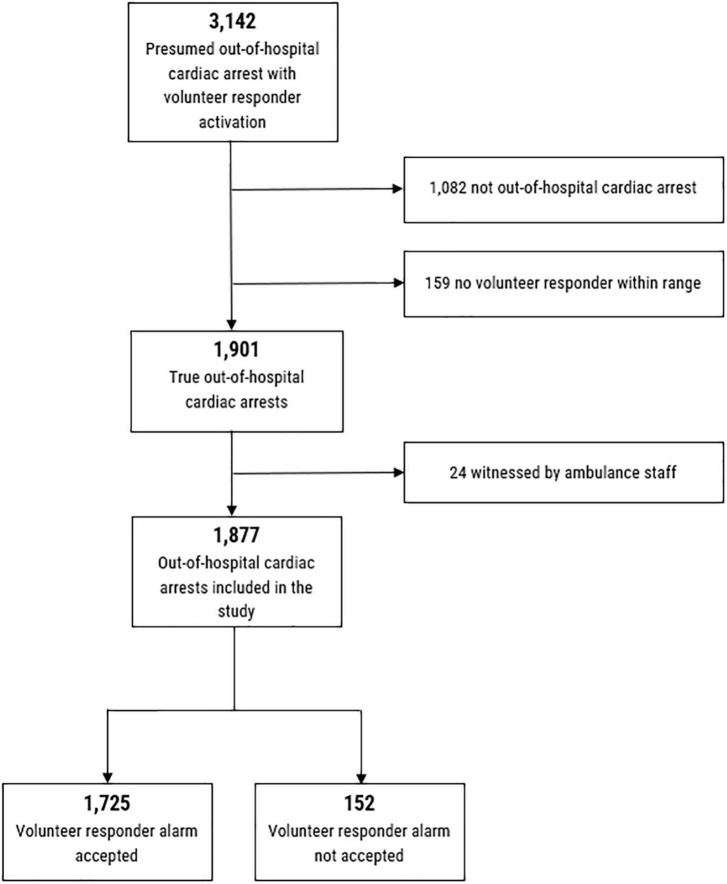
Flowchart of included patients.

We initially identified 3,142 presumed OHCAs, assessed by emergency dispatchers, with volunteer responder activation within the study period. Of these, 1,082 were verified non-OHCA, but presumably other genesis, and 24 were witnessed by ambulance staff and thus excluded from further analysis. This resulted in a study population of 1,877 OHCAs with volunteer responder activation, Figure 1. Of these, 1,725 (91.9%) OHCAs had at least one volunteer responder accepting the alarm (classified as accepted). Of these, 1,392 (80%) answered the question about arrival at the scene whereof 1,388 (99,7%) reported successful arrival at scene. Further, 1,355 (79%) answered the question about arrival before EMS whereof 883 (65%) reported arriving before EMS. In 152 OHCAs (8.1%) no volunteer responders accepted the alarm (classified as not accepted).

### Cardiac arrest characteristics

We found no difference in baseline characteristics such as sex and age and likewise, no difference was found in initial shockable heart rhythm (VF or pVT) and proportion of bystander witnessed arrests between the accepted and not-accepted groups, Table 1.

**TABLE 1:** Characteristics of population with out-of-hospital cardiac arrest and volunteer responder activation based on accept or no accept of alarm.

	Accepted (*n* = 1725)	Not-accepted (*n* = 152)	Missing (*n*)
Age, years (IQR)	73 (63−81)	73 (63−80)	47
Male sex, *n* (%)	1121 (66.7%)	100 (67.1%)	47
Witnessed arrest, *n* (%)	905 (52.6%)	81 (53.3%)	3
Initial shockable rhythm (VF/pVT), *n* (%)	486 (28.3%)	36 (23.7%)	9
Median EMS response time, min. (IQR)	7.00 (5.00−10.00)	9.00 (6.00−12.00)	53
Distance between volunteer responders and OHCA, m (IQR)	527 (298−855)	720 (384−1422)	0
Volunteer responder answered question about arrival at scene, *n* (%)	1,392 (80)	0	485
Reported arriving at OHCA site*, *n* (%)	1388 (99.7%)	0	0
Volunteer responders answered question about arrival prior to EMS, *n* (%)	1,355 (79)	0	522
Arrival prior to EMS**, *n* (%)	883 (65.3%)	0	0
Public OHCA location, *n* (%)	312 (18.1%)	17 (11.2%)	1
Population density at OHCA site, *n* (%)			1
*Low*	501 (29.1%)	74 (48.7%)	
*Intermediate*	556 (32.3%)	56 (36.8%)	
*High*	667 (38.7%)	22 (14.5%)	
Weekend, *n* (%)	474 (27.5%)	43 (27.6%)	0
Median number of activated volunteer responders based on population density, *n* (IQR)			0
*Low*	12 (5−20)	1 (1−3)	
*Intermediate*	20 (10−20)	6 (2−13.5)	
*High*	20 (20−20)	15 (10−20)	
Time of the day, *n* (%)			0
*08.00–15.59*	828 (48.0%)	54 (35.5%)	
*16.00–23.59*	602 (34.9%)	37 (24.3%)	
*00.00–07.59*	295 (17.1%)	61 (40.1%)	
EMS defibrillation, *n* (%)	498 (28.9%)	47 (30.9%)	0
Bystander CPR, *n* (%)	1543 (89.6%)	126 (82.9%)	3
Volunteer responders answering question about type of CPR performed, *n* (%)	606 (35%)	0	1,119
Reported performing compressions and ventilation, *n* (%)	213 (35)	0	
Reported performing compressions alone, *n* (%)	362 (60)	0	
Reported performing ventilations alone, *n* (%)	31 (5)	0	
Bystander defibrillation, *n* (%)	227 (13.2%)	10 (6.6%)	1
ROSC at hospital arrival, *n* (%)	471 (27.4%)	30 (19.9%)	7
30-day survival, *n* (%)	253 (15.1%)	14 (9.4%)	50

OHCA, out-of-hospital cardiac arrest; VF/pVT, ventricular fibrillation/pulseless ventricular tachycardia; EMS, emergency medical services; CPR, cardiopulmonary resuscitation; ROSC, return of spontaneous circulation. Values are median (Q1, Q3), n or n (%). *At least one volunteer responder arrives at site. **At least one volunteer responder arrives at site prior to EMS.

A longer median EMS response time (9.00 vs. 7.00 min) and longer distance from volunteer responder to OHCA site (720 vs. 527 m) were found in the not-accepted group. A larger proportion of OHCAs in the accept group occurred in public locations (18.1 vs. 11.2%) and in areas of high population density (38.7 vs. 14.5%) compared to the not-accepted group. More OHCAs where at least one volunteer responder accepted the alarm occurred during working hours (8.00 a.m.−03.59 p.m.) with no difference during evening (04.00 p.m.−11.59 p.m.) and fewer during night-time (00.00−07.59 a.m.). Still, we found more than 4 times as many incidents of accepted as not-accepted alarms during nighttime (295 vs. 61 incidents).

### Bystander interventions and outcome

**FIGURE 2:**
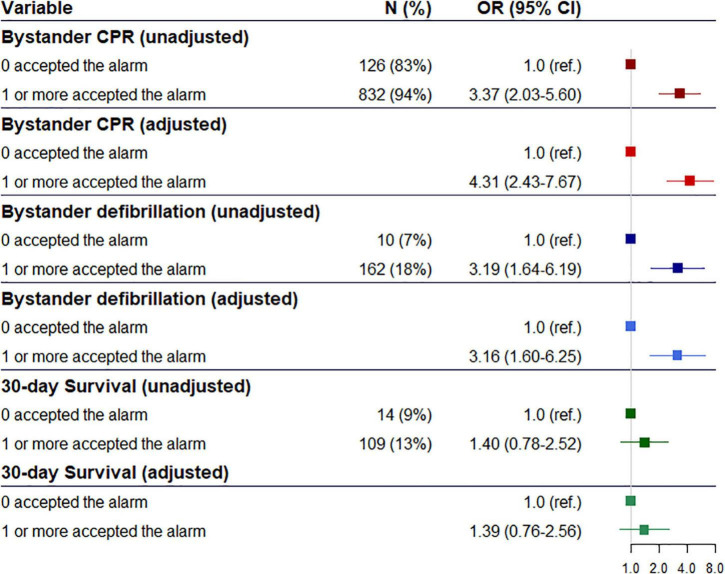
Odds ratio and adjusted odds ratio of association between alarm acceptance and bystander cardiopulmonary, bystander defibrillation and 30-day survival where at least one volunteer responder accepted the alarm and arrived before the ambulance compared to cases where no volunteer responders accepted the alarm.

**FIGURE 3:**
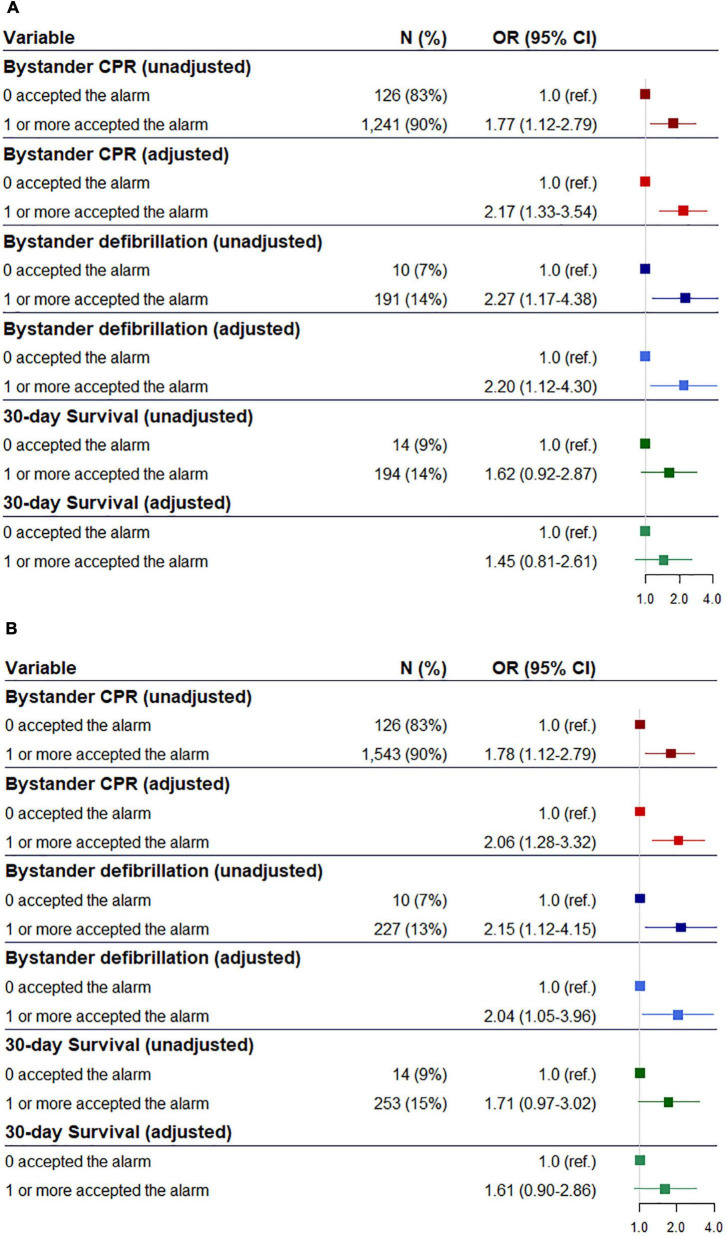
**(A)** Odds ratio and adjusted odds ratio of association between arrival at out-of-hospital cardiac arrest (OHCA) site of volunteer responder and bystander cardiopulmonary resuscitation and bystander defibrillation with alarm not accepted as reference. **(B)** Odds ratio and adjusted odds ratio of association between alarm acceptance and bystander cardiopulmonary, bystander defibrillation and 30-day survival with alarm not accepted as reference.

In our primary analysis we observed that significant more received bystander CPR [94 vs. 83%, odds ratio (OR) 3.37 95% Confidence Interval (95% CI) (2.02−5.60)] and bystander defibrillation [13 vs. 9%, 3.19 (1.64−6.19)] when a volunteer responder accepted the alarm and arrived before EMS compared to OHCAs where no volunteer responders accepted the alarm, respectively, Figure 2. After adjusting for confounders, bystander CPR [4.31 (2.43−7.67)] and defibrillation [3.16 (1.60−6.25)] remained significant. However, we observed no difference in 30-day survival, Figure 2. In the secondary analysis, we observed that significant more received bystander CPR [90 vs. 83%, 1.77 (1.12−2.79)] and bystander defibrillation [14 vs. 7%, 2.27 (1.17−4.38)] when a volunteer responder accepted the alarm and arrived at the scene of OHCA compared to cases where no volunteer responders accepted the alarm, respectively, Figure 3A. When adjusting for confounders odds ratio of bystander CPR increased [2.17 (1.33−3.54)] while the odds ratio of bystander defibrillation remained similar [2.20 (1.12−4.30)]. In the other secondary analysis of all OHCAs (irrespective of arrival status of the volunteer responders) we identified a significant association between volunteer responder acceptance of the alarm and both bystander CPR [90 vs. 83%, 1.78 (1.12−2.79)] and bystander defibrillation [13 vs. 7%, 2.15 (1.12−4.15)], with a borderline significant association in 30-day survival [15 vs. 9%, 1.71 (0.97−3.02)]. After adjusting for confounders, bystander CPR [2.06 (1.28–3.32)], and bystander defibrillation [2.04 (1.05–3.96)] were significantly associated with volunteer responder acceptance of the alarm, whereas difference in 30-day survival [1.61 (0.90–2.86)] was non-significant, Figure 3B.

Patients presenting with and initial shockable rhythm had higher survival rates (39%) when at least one volunteer responder accepted the alarm compared to cases where no one accepted the alarm (29%), Supplementary Table 1. However, these results were not statistically significant, *p* = 0.36.

## Discussion

This prospective observational study investigated whether alarm acceptance by volunteer responders in the case of OHCA was associated with increased bystander intervention and improved patient outcome and the circumstances related to the OHCA. In 9 out of 10 OHCAs we found that at least one volunteer responder accepted the alarm, with fewest alarms accepted (29.1%) in rural areas and during nighttime (17.1%) compared to urban areas (38.7%) and during daytime (48%). The adjusted odds ratio of bystander CPR and defibrillation increased fourfold and threefold, respectively, when a volunteer responder accepted the alarm and arrived before EMS compared with cases where no volunteer responders accepted the alarm. Similar, we found increased odds ratios of bystander CPR and defibrillation when volunteer responders reported arriving at scene of OHCA. Finally, when looking at all OHCAs irrespective of the volunteer responder’s arrival status we still observed increased odds ratios of bystander CPR and defibrillation when at least one volunteer responder accepted the alarm compared to OHCAs where no one accepted the alarm.

### Positive association with bystander intervention

Previous studies have shown that volunteer responder programs hold the potential to increase bystander CPR and defibrillation (5–7, 23). However, only few studies have compared the direct association between alarm acceptance and outcome. One recent study from the UK by Smith et al. found increased bystander CPR with volunteer responder acceptance compared with no volunteer responder involvement in one group (London; 70.6 vs. 65.6%) but lower bystander CPR rate in a second group (East Midlands 60 vs. 74.9%). Smith et al. also found increased bystander defibrillation (9.8 vs. 8.5%) with volunteer responder acceptance (10). The proportions from London are comparable to, but lower than our findings. This difference could be related to the very high number of accepted alarms we observe in our study with alarms acceptance in 91.9% of OHCAs with volunteer responder activation compared to respective 16% in London and 15% in East Midlands. We observed markedly increased probability of both bystander CPR and defibrillation when one volunteer responder accepted the alarm and arrived before EMS. Dispatcher assisted CPR, which is a part of the OHCA protocol in Denmark, is deemed to be one of the reasons that we still find a high proportion of bystander CPR in the not-accepted group. A high bystander engagement is also part of the culture in Denmark with high rates of bystander CPR even before implementation of the volunteer responder program (24).

A recent Dutch study by Stieglis et al. from 2020 found that 17% of all OHCAs with initial shockable rhythm were defibrillated by volunteer responders (25). Comparably, we observed bystander defibrillation in 13.2% of all OHCAs where at least one volunteer responder accepted the alarm which is twice the proportion we find with alarm not accepted (6.6%). The proportion of bystander defibrillated OHCAs increased even further when volunteer responders reported arrival at OHCA site (14%) and arrival prior to EMS (18%).

Unfortunately, we were not able to differentiate between whether CPR and defibrillation was performed by random bystanders at site or alerted volunteer responders. As public arrests were more frequent in the accept group this may have contributed to the higher occurrence of bystander intervention, as public location of arrest in itself is associated with bystander intervention as these arrests are more likely to be witnessed (26–28). As we did not find any difference in proportion of bystander witnessed arrests between our two groups why this most likely does not explain the difference in bystander CPR. It could still be a contributing factor to the difference in bystander defibrillation as publicly available AEDs generally are more accessibly in public locations (29–31).

### Time and place

An OHCA alarm was more likely to be accepted in areas of high population density. This is most likely due to availability as more volunteer responders were activated in areas of high population density, Table 1. Indeed, the 2020 study by Stieglis et al. found an association between the density of volunteer responders and the likelihood of bystander defibrillation and that this correlation was stronger in less densely populated areas where they found few volunteer responders (25).

The fact that we find a longer EMS response time in the not-accepted group could also be a result of rurality of the OHCA location as OHCAs in rural areas also were more frequent in this group. A volunteer responder study by Andelius et al. from 2020 found an association between longer EMS response time and increased bystander interventions (6). This strongly suggests a big potential to improve bystander intervention in rural areas by utilizing the potential of volunteer responder systems.

We also observed significant diurnal variations in alarm acceptance with significantly higher proportions of alarms being accepted during daytime/evening and higher proportions of not-accepted alarms during nighttime (32). However, we still found more than 4 times as many incidents of accepted as not-accepted alarms during nighttime. As OHCAs during nighttime are generally known to be associated with worse outcome (33, 34) an increased focus on volunteer responder alarm acceptance could prove beneficial (32, 35).

### Is there a potential to increase survival?

This study found a difference in 30-day survival (15.1% when at least one volunteer responder accepted the alarm vs. 9.4% where none accepted the alarm) which was statistically insignificant after adjusting for confounders [1.61 CI (0.90–2.86)]. Further, when looking only at patients presenting with an initial shockable rhythm survival increased non-significantly to 39% when at least one volunteer responder accepted the alarm compared to cases where no one accepted the alarm (29%, *p* = 0.36). This difference might indicate a potential to improve survival and that the statistical insignificance could be a result of lack of power, due to the limited number of OHCAs in the study where no volunteer responders accepted the alarm. However, the 30-day survival presented in this study is comparable to the overall survival rate after OHCA in Denmark in 2020 (14%) (13). Yet another study by Stieglis et al. from 2021 demonstrated increased 30-day survival in residential locations after implementing a volunteer responder program (7).

The UK GoodSAM system also demonstrated a difference in 30-day survival between the alarmed and not-alarmed group [London; 17.6 vs. 10.3%, 3.15 95% CI (1.19–8.36)] but interestingly, also found a difference between the groups of alarm accepted and not-accepted [3.06 95% CI (1.0.–9.03)] (10).

Currently, available studies demonstrating differences in 30-day survival are all observational studies and presenting small absolute numbers of survivors. This increases the risk of confounding and misinterpretation. Furthermore, most available studies compare volunteer responder activation with no activation which is problematic as it further increases risk of inducing both bias and confounding to the analysis as several factors related to the circumstances of the OHCA differ. To fully understand the effect of volunteer responder systems, randomized controlled trials are warranted and currently being conducted in the US/Canada (PulsePoint Study; NCT04806958) and Denmark (HeartRunner Trial; NCT03835403) (36).

### Implications of more detailed data

As demonstrated by the findings in this study, data reporting and selection of variables in volunteer responder programs have a big impact on results and the interpretation hereof. We found a clear tendency toward higher odds for bystander CPR and defibrillation in the cases where volunteer responders arrived at site and further with arrival before the EMS compared to only reporting data with respect to whether the volunteer responders accepted the alarm or not. This demonstrates why it is difficult to compare studies with different exposure and outcome variables (37) and supports the importance of having detailed data available for correct interpretation. A greater uniformity with international consensus on reported measurement variables could improve translation and sharing of knowledge (38).

### Strengths and limitations

Within the variables “arrival at site” and “arrival prior to EMS” we saw a large number of missing values as some volunteer responders have not completed the survey which should be taken into consideration. This study is limited as it is an observational study why we can only investigate associations and not causal effects. The EUROSAT degree of urbanization system (DEGURBA) (21) was used to stratify population density. This was the best tool available but arguably has some limitations such as very broadly defined subcategories which could run the risk of overlooking finer details on the population map.

## Conclusion

We observed a fourfold increased odds ratio for bystander CPR and threefold increased odds ratio for bystander defibrillation in OHCAs where volunteer responders accepted the alarm and arrived before EMS compared to cases where no volunteer responders accepted the alarm. We saw no difference in 30-day survival when volunteer responders accepted the alarm.

## Data availability statement

The original contributions presented in this study are included in the article/Supplementary material, further inquiries can be directed to fredrik.folke@regionh.dk.

## Ethics statement

Ethical review and approval was not required for the study on human participants in accordance with the local legislation and institutional requirements. Written informed consent for participation was not required for this study in accordance with the national legislation and the institutional requirements.

## Author contributions

CN: study conception and design, analysis and interpretation of data, and writing the manuscript. LA, CH, UV, EC, and CT-P: interpretation of data and revision of manuscript. AE: statistical review, analysis and interpretation of data, and revision of manuscript. FF and MG: study conception and design, analysis and interpretation of data, and revision of manuscript. All authors have read and approved the final manuscript.

## Supplementary material

SUPPLEMENTARY FIGURE 1 Directed acyclic graph showing the included variables and possible confounders for bystander cardiopulmonary resuscitation as outcome.

SUPPLEMENTARY FIGURE 2 Directed acyclic graph showing the included variables and possible confounders for bystander defibrillation as outcome.

SUPPLEMENTARY FIGURE 3 Directed acyclic graph showing the included variables and possible confounders for 30-day survival as outcome.

## References

[1] Hallstrom AP Ornato JP Weisfeldt M Travers A Christenson J McBurnie MA Public-access defibrillation and survival after out-of-hospital cardiac arrest. *N Engl J Med.* (2004) 351:637–46. 10.1056/NEJMoa040566 15306665

[2] Hasselqvist-Ax I Riva G Herlitz J Rosenqvist M Hollenberg J Nordberg P Early cardiopulmonary resuscitation in out-of-hospital cardiac arrest. *N Engl J Med.* (2015) 372:2307–15. 10.1056/NEJMoa1405796 26061835

[3] Kobayashi D Sado J Kiyohara K Kitamura T Kiguchi T Nishiyama C Public location and survival from out-of-hospital cardiac arrest in the public-access defibrillation era in Japan. *J Cardiol.* (2020) 75:97–104. 10.1016/j.jjcc.2019.06.005 31350130

[4] Panchal AR Bartos JA Cabañas JG Donnino MW Drennan IR Hirsch KG Part 3: adult basic and advanced life support: 2020 American heart association guidelines for cardiopulmonary resuscitation and emergency cardiovascular care. *Circulation.* (2020) 142:S366–468. 10.1161/CIR.0000000000000916 33081529

[5] Ringh M Rosenqvist M Hollenberg J Jonsson M Fredman D Nordberg P Mobile-phone dispatch of laypersons for CPR in out-of-hospital cardiac arrest. *N Engl J Med.* (2015) 372:2316–25. 10.1056/NEJMoa1406038 26061836

[6] Andelius L Malta HC Lippert FK Karlsson L Torp-Pedersen C Kjær Ersbøll A Smartphone activation of citizen responders to facilitate defibrillation in out-of-hospital cardiac arrest. *J Am Coll Cardiol.* (2020) 76:43–53. 10.1016/j.jacc.2020.04.073 32616162

[7] Stieglis R Zijlstra JA Riedijk F Smeekes M van der Worp WE Tijssen JGP Alert system-supported lay defibrillation and basic life-support for cardiac arrest at home. *Eur Heart J.* (2022) 43:1465–74. 10.1093/eurheartj/ehab802 34791171 PMC9009403

[8] Sarkisian L Mickley H Schakow H Gerke O Jørgensen G Larsen ML Global positioning system alerted volunteer first responders arrive before emergency medical services in more than four out of five emergency calls. *Resuscitation.* (2020) 152:170–6. 10.1016/j.resuscitation.2019.12.010 31923531

[9] Brooks SC Simmons G Worthington H Bobrow BJ Morrison LJ. The pulsepoint respond mobile device application to crowdsource basic life support for patients with out-of-hospital cardiac arrest: challenges for optimal implementation. *Resuscitation.* (2016) 98:20–6. 10.1016/j.resuscitation.2015.09.392 26475397

[10] Smith CM Lall R Fothergill RT Spaight R Perkins GD. The effect of the GoodSAM volunteer first-responder app on survival to hospital discharge following out-of-hospital cardiac arrest. *Eur Heart J Acute Cardiovasc Care.* (2022) 11:20–31. 10.1093/ehjacc/zuab103 35024801 PMC8757292

[11] Stroop R Kerner T Strickmann B Hensel M. Mobile phone-based alerting of CPR-trained volunteers simultaneously with the ambulance can reduce the resuscitation-free interval and improve outcome after out-of-hospital cardiac arrest: a German, population-based Cohort Study. *Resuscitation.* (2020) 147:57–64. 10.1016/j.resuscitation.2019.12.012 31887366

[12] Tsao CW Aday AW Almarzooq ZI Alonso A Beaton AZ Bittencourt MS Heart disease and stroke statistics—2022 update: a report from the American heart association. *Circulation.* (2022) 145:e153–639. 10.1161/CIR.0000000000001052 35078371

[13] Lippert F Jørgensen BS Rühmann B Hassager C Terkelsen CJ Torp-Pedersen C *Styregruppen for Dansk Hjertestopregister* (2020). Available online at: https://hjertestopregister.dk/wp-content/uploads/2022/06/Dansk-Hjertestopregister-Aarsrapport-2020_opdateret-jun22.pdf (accessed April 1, 2022).

[14] Valenzuela TD Roe DJ Nichol G Clark LL Spaite DW Hardman RG. Outcomes of rapid defibrillation by security officers after cardiac arrest in casinos. *N Engl J Med.* (2000) 343:1206–9. 10.1056/NEJM200010263431701 11071670

[15] Nielsen CG Andelius LC Hansen CM Blomberg SNF Christensen HC Kjølbye JS Bystander interventions and survival following out-of-hospital cardiac arrest at Copenhagen International Airport. *Resuscitation.* (2021) 162:381–7. 10.1016/j.resuscitation.2021.01.039 33577965

[16] Valeriano A Van Heer S de Champlain F Brooks SC. Crowdsourcing to save lives: a scoping review of bystander alert technologies for out-of-hospital cardiac arrest. *Resuscitation.* (2021) 158:94–121. 10.1016/j.resuscitation.2020.10.035 33188832

[17] Greif R Bhanji F Bigham BL Bray J Breckwoldt J Cheng A Education, implementation, and teams: 2020 international consensus on cardiopulmonary resuscitation and emergency cardiovascular care science with treatment recommendations. *Circulation.* (2020) 142:S222–83. 10.1161/CIR.0000000000000896 33084395

[18] Olasveengen TM Semeraro F Ristagno G Castren M Handley A Kuzovlev A European resuscitation council guidelines 2021: basic life support. *Resuscitation.* (2021) 161:98–114. 10.1016/j.resuscitation.2021.02.009 33773835

[19] *The Danish Foundation TrygFonden, You Can Save Lives*. Available online at: https://hjertestarter.dk/english/you-can-save-lives (accessed April 1, 2022).

[20] Gregers MCT Andelius L Malta Hansen C Kragh AR Torp-Pedersen C Christensen HC Activation of Citizen responders to out-of-hospital cardiac arrest during the COVID-19 outbreak in Denmark 2020. *J Am Heart Assoc.* (2022) 11:e024140. 10.1161/JAHA.121.024140 35253455 PMC9075288

[21] Dijkstra L Poelman H. *A Harmonised Definition of Cities and Rural Areas: The New Degree of Urbanisation, Regional Working Paper 2014, WP 01/2014.* Brussels: European Commission (n.d.). 28 p.

[22] R Core Team. *R: A Language and Environment for Statistical Computing.* Vienna: R Foundation for Statistical Computing (2019).

[23] Zijlstra JA Stieglis R Riedijk F Smeekes M Worp WE van der Koster RW. Local lay rescuers with AEDs, alerted by text messages, contribute to early defibrillation in a Dutch out-of-hospital cardiac arrest dispatch system. *Resuscitation.* (2014) 85:1444–9. 10.1016/j.resuscitation.2014.07.020 25132473

[24] Wissenberg M Lippert FK Folke F Weeke P Hansen CM Christensen EF Association of national initiatives to improve cardiac arrest management with rates of bystander intervention and patient survival after out-of-hospital cardiac arrest. *JAMA.* (2013) 310:1377–84. 10.1001/jama.2013.278483 24084923

[25] Stieglis R Zijlstra JA Riedijk F Smeekes M Worp WE van der Koster RW. AED and text message responders density in residential areas for rapid response in out-of-hospital cardiac arrest. *Resuscitation.* (2020) 150:170–7. 10.1016/j.resuscitation.2020.01.031 32045663

[26] Hansen SM Hansen CM Folke F Rajan S Kragholm K Ejlskov L Bystander defibrillation for out-of-hospital cardiac arrest in public vs residential locations. *JAMA Cardiol.* (2017) 2:507–14. 10.1001/jamacardio.2017.0008 28297003 PMC5814985

[27] Sondergaard KB Wissenberg M Gerds TA Rajan S Karlsson L Kragholm K Bystander cardiopulmonary resuscitation and long-term outcomes in out-of-hospital cardiac arrest according to location of arrest. *Eur Heart J.* (2019) 40:309–18. 10.1093/eurheartj/ehy687 30380021

[28] Weisfeldt ML Everson-Stewart S Sitlani C Rea T Aufderheide TP Atkins DL Ventricular tachyarrhythmias after cardiac arrest in public versus at home. *N Engl J Med.* (2011) 364:313–21. 10.1056/NEJMoa1010663 21268723 PMC3062845

[29] Karlsson L Hansen CM Wissenberg M Hansen SM Lippert FK Rajan S Automated external defibrillator accessibility is crucial for bystander defibrillation and survival: a registry-based study. *Resuscitation.* (2019) 136:30–7. 10.1016/j.resuscitation.2019.01.014 30682401

[30] Folke F Gislason GH Lippert FK Nielsen SL Weeke P Hansen ML Differences between out-of-hospital cardiac arrest in residential and public locations and implications for public-access defibrillation. *Circulation.* (2010) 122:623–30. 10.1161/CIRCULATIONAHA.109.924423 20660807

[31] Berdowski J Blom MT Bardai A Tan HL Tijssen JGP Koster RW. Impact of onsite or dispatched automated external defibrillator use on survival after out-of-hospital cardiac arrest. *Circulation.* (2011) 124:2225–32. 10.1161/CIRCULATIONAHA.110.015545 22007075

[32] Mottlau KH Andelius LC Gregersen R Malta Hansen C Folke F. Citizen responder activation in out-of-hospital cardiac arrest by time of day and day of week. *J Am Heart Assoc.* (2022) 11:e023413. 10.1161/JAHA.121.023413 35060395 PMC9238482

[33] Matsumura Y Nakada T Shinozaki K Tagami T Nomura T Tahara Y Nighttime is associated with decreased survival and resuscitation efforts for out-of-hospital cardiac arrests: a prospective observational study. *Crit Care.* (2016) 20:141. 10.1186/s13054-016-1323-4 27160587 PMC4862118

[34] Bagai A McNally BF Al-Khatib SM Myers JB Kim S Karlsson L Temporal differences in out-of-hospital cardiac arrest incidence and survival. *Circulation.* (2013) 128:2595–602. 10.1161/CIRCULATIONAHA.113.004164 24045044

[35] Stieglis R Koster RW. Volunteer responders should not be overlooked during the night. *J Am Heart Assoc.* (2022) 11:e024743. 10.1161/JAHA.121.024743 35060396 PMC9238475

[36] Folke F. *Public Access Defibrillation by Activated Citizen First-responders - The HeartRunner Trial.* Bethesda, MD: http://Clinicaltrials.gov (2020). 10.1016/j.resplu.2026.101420 PMC13495410 42630735

[37] Scquizzato T Belloni O Semeraro F Greif R Metelmann C Landoni G Dispatching citizens as first responders to out-of-hospital cardiac arrests: a systematic review and meta-analysis. *Eur J Emerg Med.* (2022) 29:163–72. 10.1097/MEJ.0000000000000915 35283448

[38] Metelmann C Metelmann B Kohnen D Brinkrolf P Andelius L Böttiger BW Smartphone-based dispatch of community first responders to out-of-hospital cardiac arrest - statements from an international consensus conference. *Scand J Trauma Resusc Emerg Med.* (2021) 29:29. 10.1186/s13049-021-00841-1 33526058 PMC7852085

