# Supplementary material for: Increased bystander intervention when volunteer responders attend out-of-hospital cardiac arrest
Source: Front Cardiovasc Med. 2022 Nov 4;9:1030843. doi: 10.3389/fcvm.2022.1030843 (PMC9672473; doi:10.3389/fcvm.2022.1030843)
Supplement: Supplementary file 1 [file Table_1.docx]

**Supplemental Table 1 – 30-day survival according volunteer responder acceptance in patients with initial shockable and non-shockable rhythm.**

| **Patients presenting with an initial shockable rhythm** | | | | |
| --- | --- | --- | --- | --- |
|  | Accepted (N=1,716) | Not-accepted (N=152) | Missing | P-value |
| Initial shockable rhythm, n (%) | 486 (28.3) | 36 (23.7) | 9 | - |
| 30-day survival, n (%) | 179 (39) | 10 (29) | 28 | 0.36 |
| **Patients presenting with an initial non-shockable rhythm** | | | | |
| Initial non-shockable rhythm, n (%) | 1,230 (71.7) | 116 (76.3) | 9 | - |
| 30-day survival, n (%) | 72 (6) | 4 (3.5) | 22 | 0.39 |
