# Supplementary figures and images for: Increased bystander intervention when volunteer responders attend out-of-hospital cardiac arrest
Source: Front Cardiovasc Med. 2022 Nov 4;9:1030843. doi: 10.3389/fcvm.2022.1030843 (PMC9672473; doi:10.3389/fcvm.2022.1030843)

**Supplementary Figure 1**

**
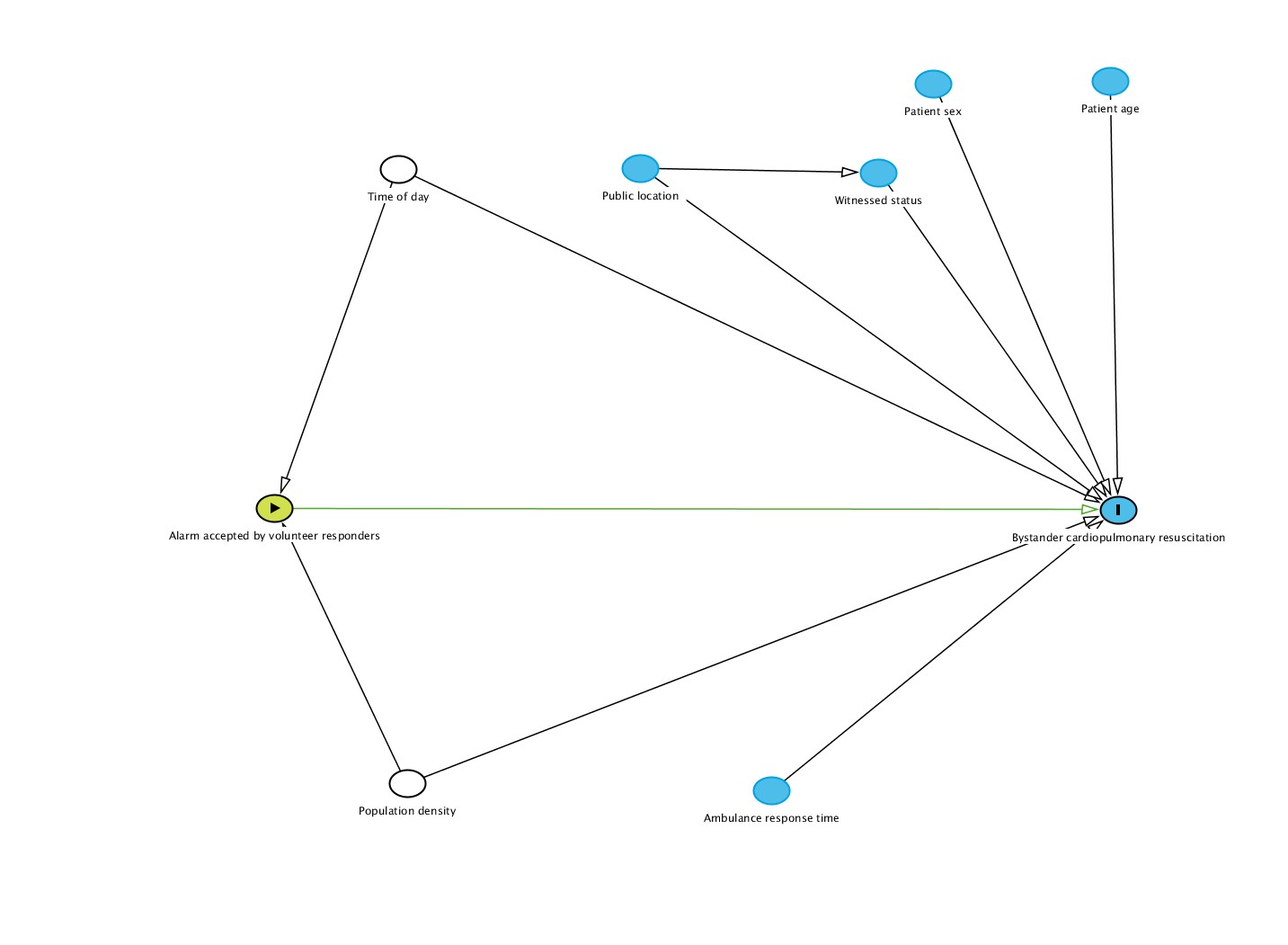
**

**Supplementary Figure 2**

**
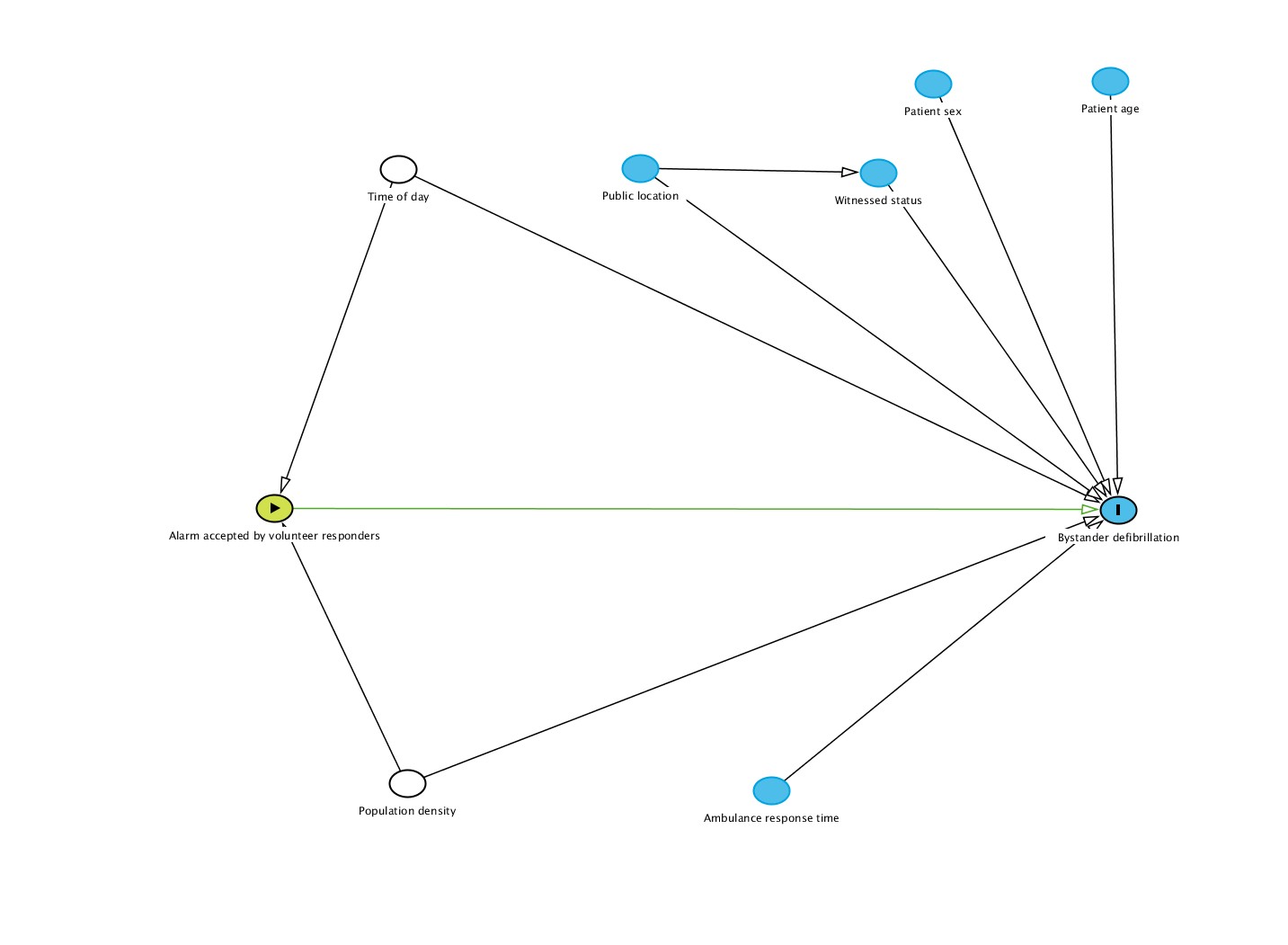
**

**Supplementary Figure 3**

**
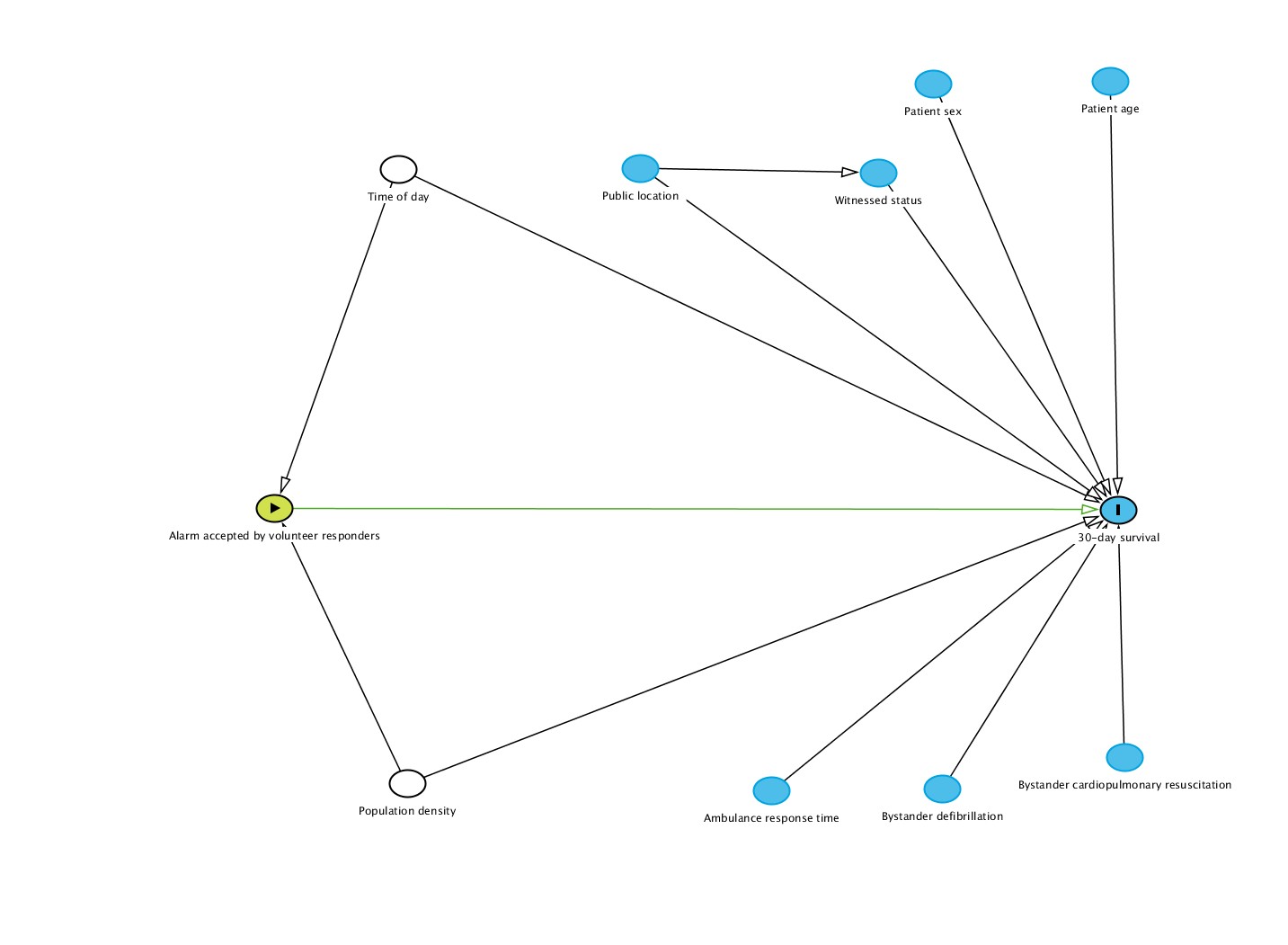
**

Supplement: SUPPLEMENTARY FIGURE 1 — Directed acyclic graph showing the included variables and possible confounders for bystander cardiopulmonary resuscitation as outcome. [file Data_Sheet_1.docx]
